# Puerarin as a multi-targeted modulator of lipid metabolism: molecular mechanisms, therapeutic potential and prospects for nutritional translation

**DOI:** 10.3389/fnut.2025.1598897

**Published:** 2025-07-18

**Authors:** Shu Ou, Qingzhi Liang, Yulin Leng, Ting Luo, Xin Xu, Hongyan Xie, Hong Gao, Jun Li, Chunguang Xie

**Affiliations:** ^1^Hospital of Chengdu University of Traditional Chinese Medicine, Chengdu, Sichuan, China; ^2^TCM Regulating Metabolic Diseases Key Laboratory of Sichuan Province, Hospital of Chengdu University of Traditional Chinese Medicine, Chengdu, Sichuan, China; ^3^Department of Endocrinology, Hospital of Chengdu University of Traditional Chinese Medicine, Chengdu, Sichuan, China; ^4^Kunming Traditional Chinese Medicine Hospital, Kunming, Yunnan, China

**Keywords:** Puerarin, natural product, lipid metabolism, molecular mechanism, nutrient transformation

## Abstract

Lipid metabolism is a dynamic and intricate process involving the uptake, synthesis, storage and catabolism of lipid compounds in the body. Its homeostasis is crucial for maintaining the health of the organism. The regulatory network of lipid metabolism homeostasis consists of several key molecules, including SREBPs, PPARs, ChREBP, FXR, LXR, AMPK, and ncRNAs. Puerarin (Pue), an isoflavone derivative, has been demonstrated to enhance lipid metabolism by modulating the aforementioned signaling cascades. Pue has found extensive application in the pharmaceutical, food, and nutraceutical industries. Considering the multi-target and multi-pathway pharmacological properties of Pue, the present study focuses on the molecular mechanism of Pue in the regulation of lipid metabolism, the spectrum of metabolic diseases, as well as the limitations of the current study and the prospect of nutritional translation. It is hoped that this study will provide a reference for the regulation of lipid homeostasis and remodeling of lipid metabolism, with the aim of optimizing clinical use and product development.

## 1 Introduction

Lipid metabolism is defined as the process of uptake, synthesis, storage, and catabolism of lipid compounds in living organisms. This complex process involves the coordination of multiple organs, and the regulation of multiple enzymes and signaling pathways, and is essential for maintaining the body’s health (1, 2). Lipid metabolism disorders are the core pathomechanism of transdiseases, which can lead to multiple systemic diseases including hyperlipidemia (3), obesity (4), type 2 diabetes (T2DM) (5), non-alcoholic fatty liver disease (NAFLD) (6), cardiovascular diseases (CVD), central nervous system (CNS) disorders (3, 7), cancer (8), osteoporosis (9), and aging (10, 11), which seriously affect human health and impose a heavy burden on global public health. Blood lipids serve as an important indicator of lipid metabolism disorders (12). Statins are the prevailing lipid-lowering pharmaceutical agents (13). However, they are susceptible to causing adverse effects like muscle symptoms, liver dysfunction, renal insufficiency, and eye disorders (14), which limits their clinical use to some extent. Further studies have shown that statin monotherapy rarely achieves guide-line-recommended low-density lipoprotein cholesterol (LDL-C) concentrations (15). Current therapeutic strategies combining statins with adjunctive agents such as ezetimibe and next-generation PCSK9 inhibitors demonstrate minimal therapeutic benefit in patients exhibiting low-to-moderate cardiovascular risk profiles (16). This limited efficacy underscores the need for personalized risk stratification and novel combinatorial approaches to optimize lipid management in this population cohort.

Pueraria mirifica, the dried root of the leguminous plant Kudzu, is widely distributed in East and Southeast Asia. Its root is rich in isoflavonoids, which have both pharmacological and nutritional activities. Pue is an isoflavone derivative isolated from the traditional Chinese medicine Pueraria lobata, which has antioxidant, anti-inflammatory, anti-tumor, immunomodulation and other biological activities (17), which is widely used in the pharmaceutical, food, and healthcare industries. A multitude of studies have demonstrated that Pue possesses the capacity to modulate lipid metabolism in a variety of targets and pathways. Moreover, the incorporation of Pue into one’s diet has been shown to yield substantial improvements in cases of lipid metabolism disorders (18). In particular, the latest research has shown that Pue reduces fat absorption in the gut through the “brain-gut axis” (19). This provides an important reference for the use of Pue as a dietary supplement in combination with other drugs to co-regulate lipid metabolism for enhanced efficacy. However, there are fewer comprehensive evaluations of the molecular mechanisms, cross-disease therapeutic potential, and nutritional translation of Puel’s regulation of lipid metabolism.

Therefore, this article reviews the regulatory network of lipid metabolism in the organism, the molecular mechanism of Pue regulation of lipid metabolism, and the spectrum of metabolic diseases, as well as the future prospect of nutritional translation. It is anticipated that this review will encourage the utilization of Pue resources and serve as a valuable reference for expanding clinical applications and translating research findings.

## 2 Molecular mechanisms of lipid metabolism

**FIGURE 1:**
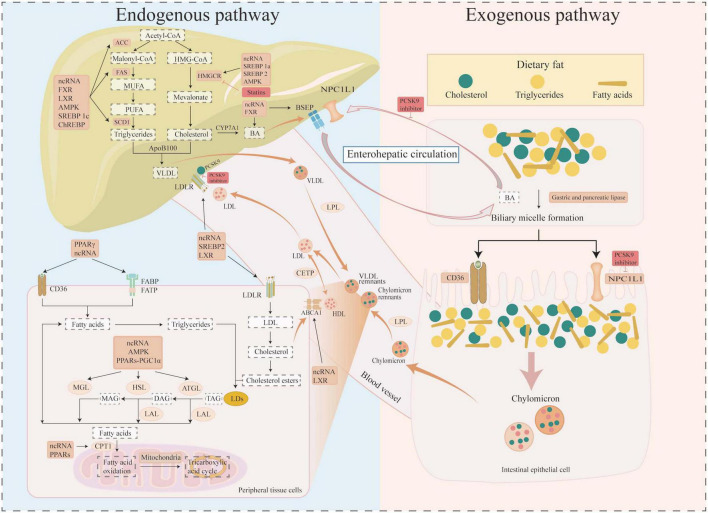
Molecular mechanisms of lipid metabolism. Diagram illustrating lipid metabolism pathways. The left section details the endogenous pathway involving liver processes, showing the uptake, synthesis, storage, and catabolism of lipid compounds such as cholesterol and triglycerides. The right section depicts the exogenous pathway, showing dietary fat absorption, bile acid micelle formation, and chylomicron creation in intestinal cells. Arrows indicate processes and interactions, with labels for molecules (such as SREBPs, PPARs, ChREBP, FXR, LXR, AMPK, and ncRNAs) and inhibitors (such as statins and PCSK9 inhibition) involved. The pathways are interconnected through enterohepatic circulation.

Lipid compounds are essential metabolites for the human body and are broadly divided into fatty acids (FAs), phospholipids (glycerophospholipids, sphingolipids) and neutral lipids [triglycerides (TGs), cholesteryl esters (CE)] (11), and their main roles include structural components, regulation of energy metabolism, and signal transduction (1). The increase in intracellular FA levels is achieved through two pathways: exogenous and endogenous (Figure 1). The exogenous pathway is primarily the digestion and absorption of lipids in the small intestine, while the endogenous pathway includes lipid uptake by tissue cells, lipid biosynthesis, lipid storage, and degradation. The process of bile micelle formation from dietary TG and cholesterol is facilitated by the actions of gastric and pancreatic lipases, as well as bile acid salts. Intestinal epithelial cells play a pivotal role in the active absorption of lipids through proteins such as cluster of differentiation 36 (CD36) and Niemann–pick C1-like 1 protein (NPC1L1). These proteins subsequently combine to form chyme particles, which then enter the circulation. It has been established that cells obtain circulating lipids primarily via CD36, fatty acid binding protein (FABP), fatty acid transporter protein (FATP), and low density lipoprotein receptor (LDLR) (1). The liver is the primary organ for *de novo* lipogenesis (DNL), and acetyl coenzyme A is a common substrate for FA and cholesterol synthesis (20). Acetyl-CoA is converted to malonyl-CoA by acetyl-CoA carboxylase (ACC). Subsequently, saturated FAs are synthesized by fatty acid synthase (FAS). The carbon chain undergoes a gradual extension and desaturation process by stearoyl-CoA desaturase 1 (SCD1) and other desaturases. The final synthesized fat is stored in cell lipid droplets (LDs) in the form of triglycerides (TAG) (11). Cholesterol biosynthesis is dominated by the rate-limiting enzyme 3-hydroxy-3-methylglutaryl-coenzyme A reductase (HMGCR) and is ultimately stored as cholesteryl esters in LDs with TAG (21, 22). Degradation of intracellular FAs is transported into mitochondria for beta oxidation, the tricarboxylic acid cycle for energy production, via carnitine palmitoyl transferase 1 (CPT1), the rate-limiting enzyme for fatty acid mitochondrial beta-oxidation. Cholesterol from peripheral tissue cells is transported from peripheral cells to the liver via high-density lipoprotein (HDL)-mediated reverse cholesterol transport (RCT) after ATP-binding cassette transporter protein (ABCA1, ABCG1, etc.) mediated cholesterol efflux (23). Cholesterol is converted to bile acids (BA) by the cytochrome P450 enzyme cholesterol 7α-hydroxylase (CYP7A1), which is subsequently actively exported from the liver via the bile salt efflux pump (BSEP)/ABCB11, and either circulated through the enterohepatic cycle or excreted in the feces (24). In addition, research has shown that extracellular vesicles, such as exosomes and microbubbles, provide an additional mechanism for cholesterol excretion outside the cell (22). Thus, lipid dynamic homeostasis involves sophisticated regulation of uptake, synthesis, storage, and catabolism, and its core network consists of several key molecules and pathways, which are analyzed in detail in the following sections.

### 2.1 Sterol regulatory element binding proteins (SREBPs)

The SREBP family comprises three major isoforms: SREBP-1a, SREBP-1c, and SREBP-2. While SREBP mRNAs are ubiquitously expressed across tissues, their abundance and functional dominance exhibit significant tissue specificity. For instance, hepatic SREBP-1c expression surpasses SREBP-1a levels by approximately tenfold (25). Functionally, SREBP-1a primarily regulates both fatty acid and cholesterol biosynthesis, acting as a master transcriptional activator of rate-limiting enzymes such as ACC and HMGCR. In contrast, SREBP-1c serves as the principal regulator of fatty acid synthesis and energy storage, directly controlling the expression of key lipogenic enzymes including ACC, FAS, and SCD1. SREBP-2 operates with high specificity in cholesterol homeostasis, governing the transcriptional activation of HMGCR and LDLR (26). Beyond their canonical roles in lipid metabolism, SREBPs function as critical signaling hubs integrating diverse biological processes, including reactive oxygen species (ROS) generation, endoplasmic reticulum stress responses, autophagy regulation, and apoptosis modulation (27, 28).

### 2.2 Peroxisome proliferator-activated receptors (PPARs)

The PPAR family comprises three distinct subtypes: PPARα, PPARβ/δ, and PPARγ. PPARα is predominantly expressed in brown adipose tissue, liver, heart, kidneys, and skeletal muscle. It coordinates lipid β-oxidation through synergistic interactions with PGC-1α, regulating key lipolytic enzymes including adipose triglyceride lipase (ATGL), hormone-sensitive lipase (HSL), and monoacylglycerol lipase (MGL). Additionally, PPARα activates thermogenic gene programs, notably upregulating uncoupling protein 1 expression to enhance energy expenditure. PPARγ, the most abundant isoform in adipose tissue, serves as a master regulator of white and brown adipocyte differentiation. It promotes lipid uptake by modulating lipid transporters CD36, FABPs, and FATPs, while stimulating lipogenesis through transcriptional activation of lipogenic enzymes such as lipoprotein lipase (LPL) (29). PPARβ/δ exhibits high activity in skeletal muscle, where it cooperates with PGC-1α to induce the expression of fatty acid catabolic enzymes and thermogenic regulators, thereby enhancing lipid oxidative metabolism and heat production (30).

### 2.3 Carbohydrate response element binding protein (ChREBP)

ChREBP is a transcription factor that promotes lipogenic gene expression by sensing carbohydrates and is a hub for hepatic lipid synthesis (31). It is predominantly expressed in metabolically active tissues including the liver, intestine, and adipose tissue, where it orchestrates hepatic lipid biosynthesis through three distinct mechanisms: first, it regulates acetyl-CoA production via the pentose phosphate pathway and glycolysis-derived citrate cleavage. The second is direct transcriptional activation of the rate-limiting enzymes in fatty acid synthesis- ACC, FAS, and SCD1-thereby facilitating fatty acid synthesis, elongation, and desaturation. Third, it upregulates the expression of microsomal triglyceride transfer protein, which promotes the formation of very low-density lipoprotein (VLDL) particles. Notably, ChREBP exhibits functional crosstalk with PPARα, creating a metabolic switch that coordinates lipid anabolism and catabolism in response to nutritional status (32).

### 2.4 Farnesoid X receptor (FXR)

The nuclear receptor superfamily member, FXR, exists as four principal human isoforms (FXRα1-α4), with predominant hepatic and intestinal expression. Functioning as a monomer or heterodimer, FXR orchestrates transcriptional programs governing BA homeostasis and lipid metabolism through three primary mechanisms: (1) Suppression of bile acid synthesis via CYP7A1 downregulation; (2) Inhibition of cholesterol biosynthesis through Insig-2 activation; (3) Coordination of enterohepatic circulation by regulating BSEP/ABCB11 and intestinal bile acid-binding protein (33). Notably, hepatic FXRα2 exerts isoform-specific functions through selective binding to ER-2 response elements (34). Beyond its established roles, FXR modulates adipogenesis by repressing SREBP-1c expression (35), positioning it as a therapeutic target for metabolic disorders, hepatobiliary malignancies, and gastrointestinal cancers (36).

### 2.5 Liver X receptor (LXR)

LXR, a pivotal member of the nuclear receptor superfamily, serves as a master coordinator of hepatic metabolic homeostasis. Its regulatory functions encompass two principal mechanisms: (1) promoting cellular cholesterol efflux through transcriptional activation of ATP-binding cassette transporters ABCA1 and ABCG1, and (2) modulating LDLR-dependent cholesterol uptake via induction of inducible degrader of LDLR (IDOL)-mediated receptor degradation, operating independently of the canonical SREBP pathway (37). Furthermore, LXR exerts cross-regulation of lipid metabolism by transcriptionally activating SREBP-1c (38), thereby bridging cholesterol homeostasis with fatty acid biosynthesis.

### 2.6 AMP activated protein kinase (AMPK)

AMPK serves as a central energy sensor orchestrating systemic metabolic homeostasis through phosphorylation-dependent regulation of key metabolic nodes. This evolutionarily conserved kinase exerts multifaceted control over lipid metabolism via three principal mechanisms: (1) suppression of DNL through inhibitory phosphorylation of ACC1/ACC2 (39) and HMGCR (40), effectively blocking FA and cholesterol biosynthesis; (2) enhancement of lipolytic capacity via activation of ATGL phosphorylation, driving fatty acid β-oxidation (41); and (3) transcriptional regulation through phosphorylation-mediated inhibition of lipogenic transcription factors SREBP-1c (42) and ChREBP (43). By integrating glycolytic flux, mitochondrial energetics, and lipid storage/oxidation programs, AMPK maintains cellular energy equilibrium while preventing ectopic lipid accumulation (44).

### 2.7 Non-coding RNAs (ncRNAs)

ncRNAs, primarily encompassing microRNAs (miRNAs), long non-coding RNAs (lncRNAs), and circular RNAs (circRNAs), lack protein-coding capacity but critically regulate lipid metabolism by modulating the expression of related genes through transcriptional, post-transcriptional, and post-translational mechanisms. miRNAs primarily function post-transcriptionally, directly targeting key enzymes and regulators (e.g., ACC, FAS, LDLR, HMGCR, ABCA1, CYP7A1) to inhibit FA synthesis and modulate cholesterol homeostasis. They also indirectly regulate lipid metabolism through core regulators like SREBPs, PGC1α, AMPK, and LXR. Notably, miR-33 is a key regulator of cholesterol homeostasis (45, 46). lncRNAs employ more complex mechanisms (e.g., signaling, scaffolding, decoying, enhancer, or guide functions) to bidirectionally regulate target genes across transcriptional, post-transcriptional, and epigenetic levels. They target central factors (e.g., ACC, FAS, SCD1, LDLR, ABCA1, ChREBP, SREBPs, AMPK, LXR, FXR) to inhibit lipid synthesis, promote cholesterol uptake, facilitate RCT and HDL synthesis, and enhance BA synthesis, typically yielding net beneficial effects. Conversely, lncRNAs can negatively regulate factors including CD36, HMGCR, ABCA1, and SREBPs. Additionally, lncRNAs modulate lipid metabolism by acting as miRNA sponges to sequester miRNAs (45, 47). Similarly, circRNAs can also function as miRNA sponges, adsorbing miRNAs to regulate key lipid metabolism molecules (e.g., FAS, PPARs, AMPK, SREBPs), thereby forming an antagonistic regulatory network with miRNAs (48, 49). Notably, HDL biogenesis is regulated by both miRNAs and lncRNAs, while HDL particles themselves serve as carriers for these ncRNAs (50, 51).

## 3 Pue’s multidimensional regulatory mechanisms

**FIGURE 2:**
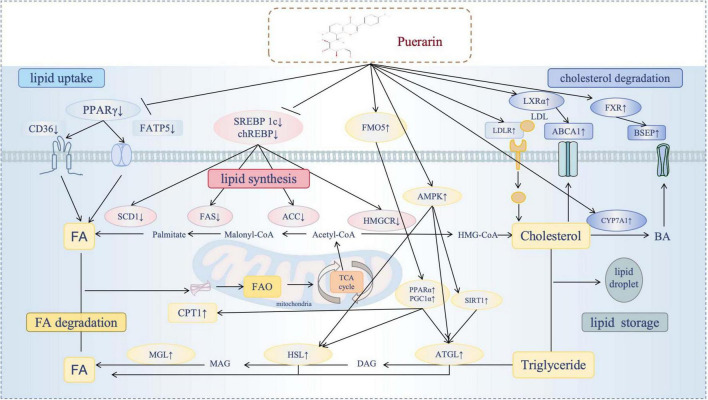
Puerarin’s multidimensional regulatory mechanisms. Diagram illustrating the effects of puerarin on lipid metabolism pathways. It shows the impact of puerarin on key lipid processes, including the reduction of lipid uptake, the inhibition of lipid synthesis, and the promotion of lipid degradation(enhancement of FA degradation and enhancement of cholesterol degradation). Puerarin modulates specific regulatory molecules (SREBPs, PPARs, ChREBP, FXR, LXR, AMPK), exerting either positive or negative effects. The flow of fatty acids, cholesterol and triglycerides was reflected in different processes.

A substantial body of research has validated the positive impacts of Pue on lipid metabolism (Figure 2), including the reduction of lipid uptake, the inhibition of lipid synthesis, and the promotion of lipid degradation. The following discussion will focus on the main molecular mechanisms by which Pue modulates lipid metabolism.

### 3.1 The inhibition of lipid uptake

Cellular FA uptake is a key regulatory node in systemic lipid homeostasis. The scavenger receptor CD36 coordinates transmembrane fatty acid transport through dynamic plasma membrane-organelle transport (52). Pharmacological studies have shown that Pue exerts a multitissue lipid-lowering effect through the coordinated regulation of FA transporters. In cardiomyocytes, Pue inhibits Na^+^/K^+^-ATPase-driven expression of CD36, which reduces FA uptake and ameliorates cardiac steatosis in *in vitro* and *in vivo* models (53). In diabetic skeletal muscle, Pue attenuates CD36 membrane translocation while increasing mitochondrial β-oxidizing capacity, thereby reducing lipid accumulation in myocytes (54). Liver studies have shown that Pue has a dual inhibitory effect on CD36 and fatty acid transporter protein 5 (FATP5), which significantly reduces lipid deposition associated with NAFLD (55). Mechanistically, Pue disrupted the PPARγ-CD36 signaling axis and effectively counteracted environmental toxicant (bisphenol S)-induced lipid deposition in C57BL/6J mice (56).

### 3.2 The inhibition of lipid synthesis

DNL, a central metabolic pathway in hepatic and adipose tissues, is dynamically regulated by nutrient-hormonal crosstalk involving insulin, glucagon, and glucocorticoids. These endocrine signals converge on transcriptional activation of core lipogenic regulators—SREBP-1c and ChREBP—through kinase-mediated signaling cascades (2). Mechanistic studies reveal that Puel exerts potent anti-lipogenic effects via multi-target suppression of the DNL machinery: Transcriptional downregulation of master regulators SREBP-1c and ChREBP; Concomitant inhibition of rate-limiting enzymes including ACC, FAS, SCD1, and HMGCR. Functional validation across experimental models demonstrating attenuated lipid accumulation in both murine hepatocytes and human HepG2 cell lines (55, 57–59).

### 3.3 The promotion of lipid degradation

#### 3.3.1 The promotion of FA degradation

Free fatty acid (FFA) β-oxidation serves as a critical pathway for maintaining lipid homeostasis. Studies have demonstrated that Pue exerts regulatory effects through multiple mechanisms. In C57BL/6J mice, Pue ameliorates bisphenol S-induced lipid accumulation by activating PPARα and CPT, thereby promoting lipidolysis (56). Through AMPK-mediated pathways, Pue suppresses PPARγ activity while enhancing HSL function, effectively improving lipid metabolism disorders (60). In HepG2 hepatocytes, Pue activates the GPER/CaMKKβ, CaMKII/CREB/SIRT1 signaling cascade, resulting in increased ATGL activity and enhanced lipid degradation (61). In high-fat/high-glucose-stimulated AML12 hepatocytes, Pue upregulates PGC-1α and SIRT1 expression, synergistically enhancing β-oxidation capacity (57). Recent evidence further identifies flavin-containing monooxygenase 5 (FMO5)/PPARα signaling as a critical pathway for Pue-induced PGC-1α and CPT1a upregulation, establishing FMO5 as a novel therapeutic node for lipid catabolism (62).

#### 3.3.2 The promotion of cholesterol degradation

Pue orchestrates systemic cholesterol clearance through two complementary mechanisms: reverse cholesterol transport and biliary efflux Potentiation. In THP-1 macrophage-derived foam cells, Pue activates the AMPK-PPARγ- LXRα axis, increasing ATP-binding cassette subfamily A member 1 (ABCA1) expression to facilitate cholesterol efflux (63). Pue acts as a FXR agonist, upregulating BSEP/ABCB11 expression to enhance BA excretion (57).

## 4 Cross-disease therapeutic applications of Pue

**FIGURE 3:**
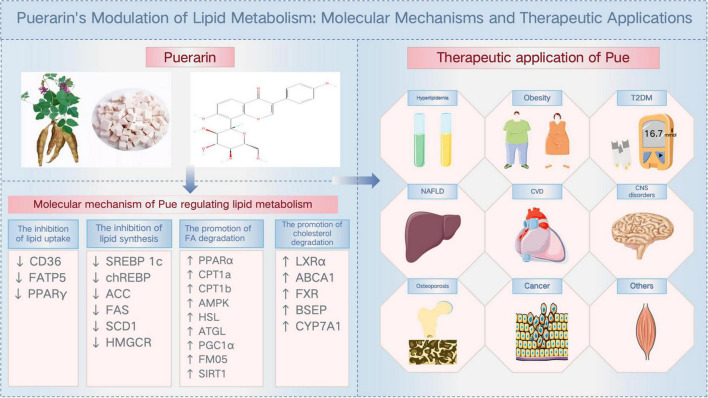
Puerarin’s modulation of lipid metabolism: molecular mechanisms and therapeutic applications.

As a natural active monomer derived from traditional Chinese medicine, Pue has a favorable safety profile and exhibits potent lipid metabolism regulation through multi-targets and multi-pathways (Figure 3; Table 1). This means that Pue has the potential to be a superior lipid-modulating drug to single-target inhibitors, which is why Pue is receiving increasing attention across disease therapies.

**TABLE 1:** Cross-disease therapeutic applications of Pue.

Disease	Animal/Cell model	The way of modeling	Dosage and duration	Mode of administration	Described effects	Pathways	References
Hyperlipidemia	Male Wistar rats	Pb	200 mg/kg/d; 75 days	Gavage	↓: ALT, AST, ROS, MDA, TC, TG, LDL, HMGCR ↑: HDL, CYP7A1, LDL-R	–	(65)
	Male ICR mice	Carbon tetrachloride	200 and 400 mg/kg/d; 8 weeks	Intragastric administration	↓: ALT, AST, TC, TG, LDL, p-JNK, p-c-Jun ↑: CYP7A1, HDL	JNK/c-Jun/CYP7A1	(66)
	Male BALB/c mice	STZ (150 mg/kg)	20, 40, and 80 mg/kg/d; 14 days	Intragastric administration	↓: Body weight, FBG, TC, TG, LDL-C ↑: HDL-C, FNS, IRS-1, IGF-1, InsR, PPARα	PPARα	(67)
	HepG2 cells	10 mM glucose, 15 mM fructose, and 0.5 mM of FFA	75 and 150 μM; 24 h	–	↓: TG, ACC, SREBP 1c, FAS, SCD-1, HMGCR ↑: AMPK	AMPK	(58)
	Male Sprague–Dawley rats	HFFD	0.2%; 16 weeks	Oral administration	↓: Liver fat indices, Perirenal fat indices, Epididymal fat indices, Retroperitoneal fat indices, TG, TC, LDL-C, MDA ↑: HDL-C, SOD, AMPK	AMPK	(58)
	Female and male C57BL/6J mice	–	0.05% and 0.1%; 3 weeks	–	↓: TC, TG, HMGCR ↑: CYP7A1, LDL-R	–	(68)
	HepG2 cells	–	50, 100, and 200 μM; 24 h	–	↓: HMGCR ↑: LDL-R, SOD	–	(68)
Obesity	Male Sprague–Dawley rats	HFD + STZ (35mg/kg)	100 mg/kg/d; 4 weeks	Intraperitoneal injection	↓: FFA, TG, MDA, CD36 ↑: p-AMPK, p-ACC, CPT1b, SIRT 1, PGC 1α, PPAR-δ, LCAD, ACOX 1, UCP2, UCP3	–	(54)
	Male Sprague–Dawley rats	HFFD	0.2%; 16 weeks	Oral administration	↓: Liver fat indices, Perirenal fat indices, Epididymal fat indices, Retroperitoneal fat indices, TG, TC, LDL-C, MDA ↑: HDL-C, SOD, AMPK	AMPK	(58)
	Female ICR mice	HFD	0.2, 0.4, and 0.8%; 12 weeks	Oral administration	↓: TC, TG, FAS, PPARγ2 ↑: AMPK, p-AMPK, HSL	–	(60)
	Male C5BL/6 mice	HFD	100 and 300 mg/kg/d; 16 weeks	Gavage	↓: Body weight, Glucose, Insulin ↑: MW to BW ratios, Cross sectional areas in muscle, p-AMPK, PGC 1α	PGC 1α	(71)
	C2C12 cells	–	10 and 20 μM; 24 h	–	↑: p-AMPK, p-ACC, PGC 1α	PGC 1α	(71)
	Male C57BL/6 mice	HFD	100 mg/kg/d; 4 weeks	Oral administration	↓: TG, FFA, ALT, CD36, SREBP 1c, ACC, FAS, SCD-1, NLRP 3, TNFα, IFN-γ, MCP-1, IL-1β, CYP7A1, Firmicutes/Bacteroidetes ratio ↑: FXR, BSEP, Shannon and Chao1’s diversity index	FXR	(57)
	AML-12 cells	HFFA	10, 30, and 100 μM; 24 h		↓: SREBP 1, NF-κB, ROS, CYP7A 1, TNFα, IFN-γ, MCP-1, IL-1β ↑: FXR, BSEP, SIRT1, PGC 1α, UCP 1	FXR	(57)
	Sprague–Dawley rats	Low-protein diet	50 mg/kg/d; 2 weeks	Intraperitoneal injection	↓: HFF	–	(72)
	Male C57BL/6J mice	HFD	37.3 μg/kg/d; 7 days	Intraperitoneal injection	↓: DMV activity, TG, body weight, jejunal fat absorption, jejunal TG content, length of microvilli ↑: total fecal lipid content, fecal fat excretion	–	(19)
T2DM	Male Kunming mice	STZ (75 mg/kg)	40, 80, and 160 mg/kg/d; 8 weeks	Gavage	↓: FBG, IRI, TG, TC, LDL-C, MDA, mTOR, NF-κB, PA, LysoPC ↑: HDL-C, SOD, AMPK, PPARγ, PC, PE	AMPK-mTOR, PPARγ-NF-κB	(74)
	Male Sprague–Dawley rats	HFD + STZ (35 mg/kg)	100 mg/kg/d; 4 weeks	Intraperitoneal injection	↓: FFA, TG, MDA, CD36 ↑: p-AMPK, p-ACC, CPT1b, SIRT 1, PGC 1α, PPAR-δ, LCAD, ACOX 1, UCP2, UCP3	–	(54)
	Rat L6 skeletal muscle cells	Palmitate (0.75 mM)	0.3 mM; 24 h	–	↓: FFA, CD36 ↑: p-ACC, p-AKT	–	(54)
	Male C57BL/6J mice	HFD + STZ (100 mg/kg)	50, 100, and 200 mg/kg/d; 8 weeks	Gavage	↓: FBG, TG, TC, Fetuin B, ACC ↑: AMPK	AMPK/ACC	(75)
NAFLD	Male Sprague–Dawley rats	HFFD	0.1% and 0.2%; 20 weeks	Oral administration	↓: TG, FFA, AST, ALT, MDA, IL-1β, IL-6, TNF-α, SREBF1, ChREBP, FAS, PLIN2, CD36, FATP5, ApoB100 ↑: VLDL, SOD, CAT, GSH-Px, CPT 1α, ATGL	–	(55)
	Male C57BL/6 mice	HFD	200 and 300 mg/kg	Intragastric administration	↓: ALT, MDA ↑: Nrf 2, SOD, GSH-Px, CPT 1α, PPARα, PGC 1α, FMO5	FMO5/PPARα	(62)
	AML-12 cells	PA (500 μM)	10, 20, 40, and 80 μM; 24 h	–	↑: PPARα	FMO5/PPARα	(62)
	HepG2 cells	OA	25, 50, and 100 μ; 24 h	–	↓: TC, TG, SREBP 1, FAS ↑: PPARα, p-AMPK	–	(59)
CVD	Male Sprague–Dawley rats	High cholesterol diet (HCD)	300 mg/kg/d; 4 weeks	Oral administration	↓: TC, TG, LDL-C ↑: CYP7A1	–	(64)
	Male white rabbits	HFD	0.1, 0.2, and 0.4 g/kg/d; 90 days	Intragastric administration	↓: TC, TG, PCNA, PDGF-A ↑: LDL-C	–	(80)
	ApoE−⁣/⁣− mice	HFD	100, 200, and 300 μM; 12 weeks.	Oral administration	↓: IL-1β, IL-6, TNF-α, ICAM-1, VCAM-1, TMA, TMAO, *P. copri*	–	(81)
	Human THP1 monocytes	PMA + oxLDL	25, 50, and 100 μg/mL; 24 h	–	↑: p-AMPK, PPARγ, LXR-α, ABCA1	AMPK-PPARγ-LXR-α-ABCA1	(63)
	Human THP1 monocytes	oxLDL	10, 50, and 100 μg/mL; 24 h	–	↓: CD36, TNFα, IL 1β, TLR 4, p-Iκ Bα/Iκ Bα, oil red intensity, early apoptotic cells of macrophages	–	(83)
	Murine RAW264.7 macrophages	oxLDL	10 and 50 μM; 24 h	–	↓: Txnip, ROS, TC, SR-A, Lox-1 ↑: Nrf2, PERK, Trx1, TrxR1	PERK/Nrf2/Trx1	(84)
	C57BL/6 mice	HFD	100 mg/kg/d; 12 weeks	–	↓: Txnip, ROS, SR-A, Lox-1 ↑: Nrf2, PERK, ATF4, Trx1, TrxR1	PERK/Nrf2/Trx1	(84)
	Female pathogen-free Sprague–Dawley rats	Bilateral OVX + AAC	50 mg/kg/d; 8 weeks	Intraperitoneal injection	↓: HW/BW, HW/TL, LVPW, LVAW ↑: LVEF, LVFS, PPARα, PGC 1α, PGC 1β, CPT 1a, CPT1b	PPARα/PGC-1	(85)
	Neonatal rat cardiomyocytes	Ang II	100 μM; 48 h	–	↓: NEFAs ↑: PPARα, PGC 1α, PGC 1β, CPT 1a, CPT1b	PPARα/PGC-1	(85)
	Primary cardiomyocytes	High glucose (HG)	1, 3, 5, 10, and 15 mM; 24 h	–	↓: CD36, CRP, IL-1b, Apoptotic cell proportion ↑: Na^+^-K^+^-ATPase acitivity	Na^+^-K^+^-ATPase	(53)
	Lipotoxic cardiomyopathy mice	–	90 mg/kg/d; 12 weeks	Intravenous injection	↓: CD36, CRP, IL-1b, Apoptotic cell proportion ↑: Na^+^-K^+^-ATPase activity	Na^+^-K^+^-ATPase	(53)
CNS disorders	Male Sprague–Dawley rats	HFHS	5% PUE; 12 weeks	Oral administration	↓: TC, TG, LDL-C, SBP, DBP, MAP, AI, LDH, CK, LVAW, LVID, EDV, α-SMA, BNP, ANP, CSA, Collagen I, NeuN-positive cells number in the hippocampus and cortex, CRP ↑: HDL-C, E/A	–	(18)
	Male Sprague–Dawley rats	HFD	30, 60, and 120 mg/kg/d;7 days	–	↓: TLR 4, IL-6, TNFα, cPLA 2, COX-2, PGE2 ↑: IL-10	TLR4/cPLA2/COX-2	(91)
	Male ICR mice	Chronic unpredictable mild stress	30 and 100 mg/kg/d; 4 weeks	Intragastric administration	↓: Desulfovibrionales ↑: Sucrose preference ratio, α-diversity index, the ratio of Bacilli/Clostridia, Bacillales, Lactobacillales and Bacillales	–	(92)
Osteoporosis	Female Sprague–Dawley rats	Ovariectomy	50 and 100 mg/kg/d; 14 weeks	Oral administration	↓: TRAcP-5 b, CTX-1, BALP, Firmicutes-to-Bacteroidetes (F/B ratio), LPS, IL-1β, TNF, IL-6 ↑: BV/TV, Tb.N, Tb. Th, BMP, OPG/RANKL, acetic acid, Butyric acid, valeric acid, total SCFAs	–	(96)
	MC3T3-E1 cells	10 mM β-sodium glycerophosphate, 50 μM vitamin C, and 10 nM dexamethasone	10 μM; 48 h	–	↑: Col 1a, Runx, ALP	–	(97)
	Male C57BL/6J mice	HFD + STZ (40 mg/kg)	25 and 50 mg/kg/d; 8 weeks	Oral administration	↓: Lipid droplets fraction area, TRAcP positive are ↑: BV/TV, Tb.N, Ct. Th	–	(97)
	Female Sprague–Dawley rats	OVX	100 mg/kg/d; 14 weeks	Oral administration	↓: BMD, CTX-I, TRACP-5 b, RANKL, TG, TC, LDL-C, PPARγ, the ratio of adipocytes versus osteoblasts ↑: Tb. Sp, OPG, BMP, PICP, Wnt 3a, β-catenin, Runx 2	–	(98)
Cancer	MCF-7/adr	Adriamycin	100 μ; 24 h	–	↓: CREB ↑: p-AMPK, p-ACC	–	(109)
Others	Zebrafish larvae	2% alcohol	40 μM; 36 h	–	↓: Liver gray level	–	(111)
	Wild-type AB line zebrafish	2% ethanol solution	25 μM; 48 h	–	↓: Liver gray level, TC, TG, FAS, HMGCR, IL 1β, TNFα, ACC ↑: p-AMPK	AMPKα-ACC	(112)
	Male Sprague–Dawley rats	–	150 mg/kg/d; 2 weeks	Gavage	↓: Body weight, prevotellaceae ↑: Grip strength/BW, specific twitch forces of SOL, specific tetanic forces of SOL, specific twitch forces of EDL, specific tetanic forces of EDL, CSA of type II fiber, Erysipelotrichaceae, Clostridia, Peptococcaceae, n-butyric acid, total SCFAs, ATP concentration	–	(113)
	Bone marrow stromal cells	0.09 mol/L ethanol	0.01 mg/mL; 21 days	–	↓: TG, PPARγ ↑: ALP, osteocalcin	–	(114)
	Kunming mice	46% ethanol	0.5 g/kg; 10 months	Intramuscular injection	↓: TC, TG, Empty osteocyte lacuna, largest fat cell diameter, PPARγ ↑: ALP, osteocalcin	–	(114)

### 4.1 Hyperlipidemia

Hyperlipidemia, defined as a disturbance in the balance of plasma lipids, is most frequently manifested as hypercholesterolemia. This condition is a well-established risk factor for a variety of metabolic disorders, including obesity, T2DM, CVD, NAFLD, and acute pancreatitis (3). Experimental studies demonstrate that Pue ameliorates high-cholesterol diet-induced hyperlipidemia through CYP7A1 upregulation (64). Mechanistically, Pue exerts dual regulatory effects by suppressing HMGCR activity (reducing cholesterol biosynthesis) while enhancing CYP7A1 and LDLR expression, thereby promoting cholesterol excretion and LDL clearance. This dual mechanism underlies its protective effects against lead-induced hyperlipidemia (65). Notably, the JNK/c-Jun/CYP7A1 pathway has been identified as a critical mediator of Pue’s anti-hyperlipidemic activity in carbon tetrachloride (CCl4)-induced models (66). In diabetic models, Pue shows therapeutic potential through PPARα activation in gastrocnemius muscle tissue, effectively reducing lipid accumulation in streptozotocin (STZ)-induced diabetic mice (67). Cellular studies reveal that Pue inhibits lipid deposition in HepG2 hepatocytes via AMP-activated protein kinase (AMPK)-mediated suppression of acetyl-CoA carboxylase (ACC) activity, concurrently downregulating adipogenic markers (SREBP-1c, FAS, SCD, and HMGCR) (58). Interestingly, while Pue and its glycosides consistently upregulate LDLR expression in both HepG2 cells and C57BL/6J mice, their effect on CYP7A1 exhibits model specificity–significantly enhancing mRNA levels in murine liver without affecting *in vitro* systems (68).

### 4.2 Obesity

Obesity is typically characterized by a state of energy imbalance, whereby energy intake exceeds energy expenditure, leading to excessive fat storage. Obesity is a significant risk factor for a number of diseases, including T2DM, NAFLD, CVD, and cancer (69). Current epidemiological data indicate over 600 million adults worldwide are clinically diagnosed with this condition (70). In high-fat diet/streptozotocin (HFD/STZ)-induced diabetic Sprague–Dawley rats, Pue administration significantly attenuated obesity-related metabolic derangements, manifested by reduced serum TG and FFA levels concomitant with decreased body weight (54). Notably, Pue demonstrated comparable efficacy in high-fat fructose diet-induced obese SD rats (58). Pue is also effective in modulating dyslipidemia through AMPK activation and coordinated regulation of key lipid-metabolizing enzymes: upregulating HSL while suppressing PPARγ (60). Pue enhances PGC-1α expression in skeletal muscle of HFD-fed mice, counteracting obesity-associated complications (71). Mechanistic studies further elucidate its multi-target effects: Inhibiting lipid absorption/synthesis via FXR-mediated downregulation of CD36, SREBP-1c, ACC, and FAS; Modulating bile acid homeostasis through CYP7A1 suppression and BSEP induction; Restoring gut microbiota composition, particularly Firmicutes/Bacteroidetes ratio and relative abundances of Firmicutes/Ascomycota phyla in HFD-induced obese C57BL/6 mice (57). Emerging evidence highlights Pue’s developmental programming effects–early intervention ameliorates hepatic steatosis and may mitigate adult-onset obesity in intrauterine growth restriction models (72). Recently discovered, Pue exerts central nervous system-mediated anti-obesity effects by binding to γ-aminobutyric acid type A receptor (GABA_*A*_R), thereby suppressing dorsal motor nucleus of the vagus neuronal activity. This neuroendocrine mechanism leads to jejunal microvilli shortening and consequent inhibition of intestinal fat absorption (19).

### 4.3 T2DM

T2DM, defined as a chronic metabolic disorder characterized by persistent hyperglycemia, arises from pancreatic β-cell dysfunction coupled with systemic insulin resistance. Moreover, Emerging evidence indicates that chronic intracellular lipid accumulation (lip toxicity) contributes to β-cell dysfunction through multiple molecular mechanisms, including endoplasmic reticulum stress, oxidative stress, inflammatory responses, mitochondrial dysfunction, and impaired autophagy (73). Pue reduces TC, TG, LDL, PA, and Lysophosphatidylcholine (LysoPC) levels, increase HDL, PC, and PE levels, improve glucose-lipid metabolism and reduce inflammatory damage through AMPK-mTOR and PPARγ-NF-κB signaling pathways, thus treating diabetes mellitus (74). In skeletal muscle, Pue increased AMPK, SIRT1, PGC-1α, and CPT-1β levels and promoted fatty acid oxidation, thereby preventing lipid accumulation in diabetic models (54). Concurrently, hepatic insulin resistance is improved through Fetuin B suppression, AMPK phosphorylation, and inhibition of ACC activity (75). It is noteworthy that Professor Roy Taylor, a prominent British scholar, has underscored that lipid deposition in the liver and pancreas serves as the initiating factor that precipitates diabetes mellitus (76). This provides an important reference for Pue to target the regulation of lipid metabolism in the treatment of diabetes mellitus and its complications.

### 4.4 NAFLD

NAFLD affects approximately 25.24% of the global population (77), with pathogenesis driven by hepatic lipid dysregulation through enhanced FA uptake and DNL, ultimately triggering cellular stress, inflammation, tissue remodeling, and fibrosis. Experimental evidence demonstrates that Pue can reduce the expression of FATP 5, CD36, SREBF1, ChREBP, ACC, and FAS to decrease lipid uptake and biosynthesis, and up-regulate the expression of CPT 1a, ATGL, and ApoB100 to promote the degradation of lipids, which significantly improves the accumulation of lipids in the livers of rats with NAFLD (55). Mechanistic studies reveal Pue activates the FMO5/PPARα axis to stimulate PGC1α and CPT1a expression, thereby promoting FA oxidation and reducing lipid deposition in NAFLD mice (62). *In vitro* validation using oleic acid-treated HepG2 cells confirms Pue’s multi-target action: activation of PPARα and AMPK pathways concurrently inhibits SREBP1 and FAS expression while enhancing FA oxidation, effectively counteracting lipid accumulation (59).

### 4.5 CVD

CVD is the leading cause of death worldwide. Atherosclerosis (AS), the primary CVD manifestation, results from cholesterol deposition in arterial walls, leading to plaque formation and vascular dysfunction (78). LDL critically drives AS progression (79), underscoring lipid metabolism regulation as a key therapeutic target. Pue exerts anti-atherogenic effects by reducing serum TG, TC, and LDL levels while enhancing CYP7A1 expression in hypercholesterolemic models, thereby promoting cholesterol and bile acid excretion and lowering atherogenic indices (64). *In vivo* studies demonstrate Pue’s capacity to inhibit aortic intimal thickening and plaque formation in rabbits (80). In addition, Pue alleviate AS by inhibiting the production of *Prevotella copri* (*P. copri*) as well as trimethylamine (TMA) (81), while trimethylamine-N-oxide (TMAO) relies on the gut microbiota to improve lipid metabolism by regulating reverse cholesterol transport (82). At the cellular level, Pue combats foam cell formation through dual actions: (1) activating the AMPK-PPARγ-LXRα pathway to promote ABCA1-mediated cholesterol efflux (63), and (2) downregulating CD36 expression to limit lipid uptake in human THP-1 macrophages (83). Further studies reveal Pue’s activation of the PERK/Nrf2/thioredoxin 1 (Trx1) axis reduces scavenger receptor-A (SR-A) and lectin-type oxidized LDL receptor-1 (LOX-1) expression, inhibiting macrophage lipid accumulation (84). In cardiac pathophysiology models, Pue attenuates myocardial hypertrophy via PPARα/PGC-1α-mediated upregulation of CPT1a/b, enhancing fatty acid oxidation (85). It concurrently preserves cardiac function by increasing Na+/K+-ATPase activity and suppressing CD36-mediated fatty acid uptake (53). Notably, Pue supplementation demonstrates preventive efficacy against diet-induced metabolic syndrome and CVD complications by improving glucolipid homeostasis, attenuating cardiovascular remodeling, and reducing atherogenic indices (18), positioning it as a promising nutraceutical for cardiometabolic disease prevention.

### 4.6 CNS disorders

Pue exhibits therapeutic potential for central nervous system (CNS) disorders through multimodal mechanisms (86–88). Emerging research highlights the intersection of lipid metabolic dysregulation and CNS pathophysiology (89). Hypercholesterolemia exacerbates neuroinflammation, accelerating neuronal degeneration and cognitive decline (90). Dietary supplementation with Pue attenuates the metabolic syndrome and associated neurological damage induced by a high-fat/high-sugar diet by restoring glycolipid homeostasis and improving cortical/hippocampal vascularity and neuronal density (18). In addition, Pue modulates phospholipid metabolism and attenuates inflammation associated with depression by inhibiting TLR4 and repairing the intestinal barrier (91). Gut-brain axis studies reveal Pue’s microbiota remodeling capacity in chronic stress models: Enriching beneficial taxa (Firmicutes, Lactobacillus) and Depleting pathogenic genera (Proteobacteria, Desulfovibrio) (92). These commensal microbes enhance intestinal integrity and immunomodulation through short-chain fatty acid (SCFA) production (93, 94). Notably, recent advances position cellular senescence as a therapeutic frontier for CNS disorders (95), with lipid metabolic alterations driving senescence progression (10). Pue’s dual targeting capability—modulating lipid metabolism while clearing senescent cells—presents a novel strategy for treating age-related neurological diseases.

### 4.7 Osteoporosis

Osteoporosis is characterized by low bone mass and deterioration of bone micro-structure. Pue exerts anti-osteoporotic effects by regulating the type and abundance of intestinal flora, increasing the content of SCFAs in the colon, especially acetic acid and butyric acid, maintaining the dynamic balance of the colonic mucosa, and reducing the inflammatory response (96). Pue influences gut microbial diversity by modulating Alloprevotella, Rodentibacter, Alistipes, and Fusobacterium flora, remodels gut flora, and regulates alpha-linolenic acid metabolism and glycerophospholipid metabolism, thereby inhibiting pioglitazone-mediated bone loss (97). Pue can also improve OVX-induced osteoporosis by activating the Wnt 3a/β-catenin signaling pathway, inhibiting the PPARγ signaling pathway, regulating phospholipid metabolism and the biosynthesis of PUFAs, and modulating the differentiation of bone mesenchymal stem cells to osteoblasts (98).

### 4.8 Cancer

Lipid signaling critically regulates tumor progression and microenvironment remodeling, with Pue emerging as a multi-target anticancer agent since its initial antitumor activity in colon cancer was reported in 2006 (99). Subsequent studies validate Pue’s efficacy across esophageal (100), lung (101, 102), hepatic (103), breast (104), and bladder cancers (105) through five core mechanisms: suppressing cancer cell proliferation/migration (105), inducing apoptosis (106), reprogramming glucose metabolism (107), overcoming chemoresistance, and modulating tumor immune landscapes (108). Mechanistically, Pue reverses multidrug resistance (MDR) in human breast cancer MCF-7/adr cells by upregulating AMPK and ACC, thereby inhibiting MDR1 expression (109). This finding holds particular significance given that phospholipid/cholesterol-mediated MDR pathways represent major obstacles in chemotherapy (110). Crucially, Pue’s ability to target lipid metabolic nodes—including MDR1 suppression and chemoresistance reversal—positions it as a promising candidate for developing lipid-centric anticancer strategies, offering novel therapeutic opportunities for oncology patients.

### 4.9 Others

Pue reverses alcohol-induced metabolic disturbances, including sphingolipid metabolism, resulting in hepatoprotective effects (111). In alcoholic fatty liver disease (AFLD), Pue ameliorates hepatic lipid accumulation in zebrafish larvae by suppressing fatty acid synthesis via the AMPKα-ACC pathway, while concurrently restoring sphingolipid homeostasis to exert hepatoprotective effects (112). Beyond hepatic protection, Pue counteracts sarcopenia by remodeling the gut-muscle axis: enhancing gut microbiota diversity, elevating SCFA production, and boosting ATP synthesis to improve skeletal muscle strength (113). Additionally, in alcohol-related osteopathology models, Pue reduces PPARγ expression in both murine bone marrow stromal cells and Kunming mice, effectively inhibiting bone marrow adipogenesis while preserving osteogenic differentiation capacity, thereby mitigating alcoholic osteonecrosis (114).

## 5 Clinical translation and nutritional considerations

Despite Pue’s clinical potential in metabolic regulation, its poor oral bioavailability due to low aqueous solubility and erratic lipid dispersion poses significant translational challenges. Pharmaceutical innovations have addressed these limitations through advanced delivery systems including cubic liquid crystal nanoparticles (115), chitosan/PLGA-based nanocarriers (116), long-circulating liposomes (117), and self-microemulsifying formulations (118), which collectively enhance Pue’s absorption and therapeutic efficacy. Clinically, Pue supplementation demonstrates metabolic benefits across multiple trials: oral administration (400 mg/day for 10 days) improves cardiac function in chronic heart failure patients by elevating left ventricular ejection fraction and reducing oxidized LDL levels (119); intravenous delivery (500 mg/day) ameliorates dyslipidemia and insulin resistance in coronary artery disease through TG, TC, and LDL-C reduction alongside HDL-C elevation (120); prolonged oral regimens (150 mg/day for 12 weeks) effectively lower cholesterol in polycystic ovary syndrome when combined with standard therapies (121); oral regimens (750 mg/day) effectively lower cholesterol in diabetic nephropathy cohorts when combined with standard therapies (122). Paradoxically, a short-term trial in Chinese males (18–50 years) showed no significant lipid improvement (123), potentially reflecting population-specific responses or trial design limitations. Future clinical validation should prioritize expanded demographic inclusion, gender-balanced cohorts, and standardized dosing protocols to establish robust evidence for Pue’s nutraceutical applications in lipid metabolic disorders.

It should be emphasized that intravascular administration of Pue can cause adverse reactions including drug fever, rash, nausea, vomiting, diarrhea, hepatic/renal damage, palpitations, anaphylactic shock, and hemolysis (124). Critically, hemolysis represents a key limiting factor for Pue injection’s clinical use. This hemolytic effect has been confirmed in both animal and cellular models: Rabbits receiving 25 mg/kg/day Pue developed hemolysis after 42 days (125). Furthermore, *in vitro* erythrocyte experiments demonstrated that Pue induces hemolysis in a dose- and time-dependent manner (126). Pharmacokinetic studies reveal its inhibitory effects on cytochrome P450 isozymes (CYP2B6, CYP2C9, and CYP3A4) (127), which may potentiate statin plasma concentrations–necessitating vigilant creatine kinase monitoring during coadministration. Furthermore, long-term estrogen use may increase the risk of breast cancer (128). The estrogenic properties of Pue raise potential concerns about long-term endocrine effects. Therefore, comprehensive toxicological evaluations of Pueraria mirifica derivatives are imperative to assess breast cancer risk associations and establish safe administration protocols, particularly for chronic therapeutic applications. These precautionary measures will facilitate the development of evidence-based guidelines to optimize Pue’s clinical translation while mitigating iatrogenic risks.

## 6 Summary and outlook

Lipid metabolism, a dynamically regulated process orchestrated by core molecular networks involving SREBPs, PPARs, ChREBP, FXR, LXR, AMPK, and ncRNAs, represents a critical therapeutic frontier. Unlike single-target lipid-lowering agents (statins, ezetimibe, PCSK9 inhibitors), Pue exhibits multi-target regulatory capacity, simultaneously modulating interconnected pathways to achieve synergistic therapeutic effects while minimizing adverse outcomes associated with monotarget interventions—exemplifying the systemic advantages of natural products. Nevertheless, four critical research gaps require attention:

First, while lipid storage mechanisms—particularly lipid droplet (LD) dynamics as functionally active organelles mediating fatty acid trafficking, storage, and interorganelle communication (129)—constitute essential components of lipid homeostasis, Pue’s regulatory effects on LD biogenesis/remodeling remain unexplored. Second, key lipid chaperones and transcription factors including FABPs that coordinate HSL-mediated lipolysis and PPARγ-driven adipogenesis (130), transcription factor EB (TFEB) regulating autophagy-lipid metabolism crosstalk via PGC-1α/PPARα (131), and forkhead box O1 (FOXO1) governing ATGL/LAL-mediated lipolysis and adipocyte differentiation (132) represent promising yet uninvestigated targets for Pue’s metabolic actions. Third, ncRNAs are established regulators of lipid metabolism, offering novel biomarkers and therapeutic targets for related diseases while demonstrating potential for individualized therapy in precision medicine. However, current studies on Pue have primarily focused on: miRNA-mediated pathways (including antioxidant, anti-inflammatory effects, inhibition of cellular pyroptosis, and cardioprotection) (133–136); lncRNA Anril-regulated autophagy (137); lncRNA/mRNA co-expression networks; and the role of circ_0020394 as a molecular sponge for miR-328-3p promoting apoptosis (138),while lacking specific studies on ncRNA regulation of lipid metabolism. In addition, HDL is both a regulatory target of ncRNA and a transport carrier of ncRNA, and the interaction between the two remains to be elucidated. Therefore, future studies should investigate the activity of Pue in the ncRNA-lipid metabolism axis and elucidate how the dynamic transport of HDL-ncRNA contributes to its therapeutic efficacy, thus laying the molecular foundation for individualized drug administration. Fourth, despite compelling preclinical evidence, clinical validation through multicenter randomized controlled trials evaluating Pue’s lipid-modulating efficacy and safety remains limited.

Emerging insights into Pue’s novel brain-gut axis-mediated fat absorption inhibition reveal its potential as a master metabolic regulator, suggesting combinatory therapeutic strategies with conventional lipid-lowering agents. Given lipid metabolism’s centrality in energy homeostasis and cellular physiology, elucidating Pue’s molecular interplay with intracellular lipid networks and advancing translational nutrition research will not only enable cross-disease therapeutic innovations but also optimize clinical applications and nutraceutical development.
